# The Link between the Consumer and the Innovations in Food Product Development

**DOI:** 10.3390/foods9091317

**Published:** 2020-09-18

**Authors:** Raquel P. F. Guiné, Sofia G. Florença, Maria João Barroca, Ofélia Anjos

**Affiliations:** 1 Research Centre for Natural Resources, Environment and Society (CERNAS), Polytechnic Institute of Viseu, 3504-510 Viseu, Portugal; raquelguine@esav.ipv.pt; 2 Faculty of Nutrition and Food Sciences (FCNAUP), University of Porto, 4150-180 Porto, Portugal; sofiaguine@gmail.com; 3 Polytechnic Institute of Coimbra, Coimbra College of Agriculture, Bencanta, 3045-601 Coimbra, Portugal; 4 Department Chemistry, Molecular Physical-Chemistry R&D Unit, University of Coimbra, 3004-535 Coimbra, Portugal; 5 Polytechnic Institute of Castelo Branco, 6000-084 Castelo Branco, Portugal; ofelia@ipcb.pt; 6 CEF, Forest Research Centre, School of Agriculture, University of Lisbon, 1349-017 Lisbon, Portugal; 7 CBP-BI, Plant Biotechnology Centre of Beira Interior, 6001-909 Castelo Branco, Portugal

**Keywords:** buying intention, consumer acceptance, marketing innovation, price

## Abstract

New lifestyles, higher incomes and better consumer awareness are increasing the demand for a year-round supply of innovative food products. In past decades, important developments have been achieved in areas related to food and the food industry. This review shows that factors influencing performance in new product development (NPD) are dynamic and continuously guiding project development. The data obtained by direct involvement of consumers can impact positively successful product development and enhance the company’s financial performance. The study of consumer behaviour and attitudes towards new foods encompasses multiple aspects, such as preference, choice, desire to eat certain foods, buying intentions and frequency of consumption. Additionally, both the consumers’ willingness to purchase and the willingness to pay a premium are important in NPD, launching and success.

## 1. Introduction

Today’s manufacturing companies rely much on the success of new products, and this has become critical for a healthy business performance, having in mind the present competitive and fast shifting markets [1,2].

Developing appropriate strategies for achieving successful new product development (NPD) has required increasing consideration. Attention has been given to exposing the drivers of successful new product performance while at the same time highlighting the importance of measuring that performance ensuring viable product life-cycle (PLC). Still, it has been observed that the majority (50–75%) of consumer-packaged goods do not achieve desired levels of success, in general, and this is also a reality in the case of the food industry, for which contributes some degree of food neophobia [1,2].

Presently, the food sector is considered one of the most important in the current global economy. Nevertheless, food industry or food service companies still face many challenges in managing their products and competing in the market. In fact, the food manufacturing industry has been recognized as an area with high degrees of new product failure [1,3,4,5].

**Figure 1:**
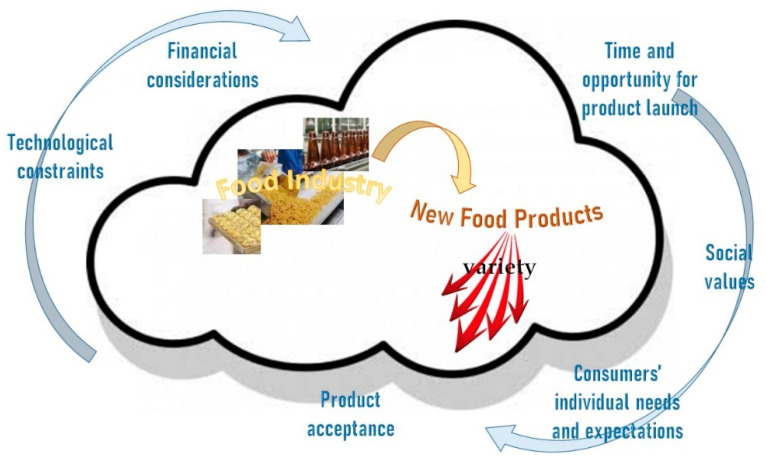
Industrial food development strategy (author’s own work).

Products aim to fulfil certain needs, which are not constant because of differences among users, constrains, usage scenarios and social values, among others. Hence, in order to meet these differences, manufacturers rely on variety as a way to target different needs and preferences (Figure 1). In this sense, it is important to clarify some concepts: Variety or assortment is defined as a number or collection of different items of a particular class of the same general kind, while variant is an instance of a class that exhibits usually slight differences from the common type. Product variety is beneficial in a way that offers potential to expand markets, with economic benefits by increasing sales’ volume and revenues. This market expansion can have two dimensions: on one hand, to reach entirely new customer segments, while on the other, being able to sell to existing customer segments more customized products repositioned as premium options. Nevertheless, this positive result is not automatic, and therefore, it must be evaluated. Variety is not necessarily always good, and more product variants may not be the best for customers when making purchase choices. It has been shown that when consumers have to choose among items in a wide assortment, frequently, they become too confused and cannot really perceive the differences between product variants and product quality. Besides, offering additional products with improved characteristics can bring increased costs from product design to production, inventory, marketing and service. Therefore, a deep evaluation must be done before making decisions about diversification of the present offer [6,7,8].

Historically, three main research perspectives in new food product development can be pointed out: (1) a technological perspective, according to which technological progress was the main driver of research and innovation in early times. Examples include technologies such as freezing or pasteurization or more recently extrusion, all technologies that were quite innovative in their own time. (2) A market-oriented perspective, according to which, back in the mid-60s, the establishment of marketing and appearance of supermarkets allied to new packaging and increased competition led to innovations in manufacture and marketing of distinguishable foods. (3) A consumer-led product development, which has more recently attracted attention to increase new products’ success [1,9].

These approaches appear relatively independent, with technological aspects and product performance traditionally studied by food scientists and consumer researchers, whereas marketing and promotion of new food products would be in the field of economics, management and marketing. Nevertheless, at present, it is demonstrated that there is a need to integrate marketing, consumer research, food design and food technology to improve new food product performance [1,9]. Attempts have been made to establish an integrated approach to food product development, combining the different subjects that altogether contribute for the positive appreciation of the product by the final consumers (ex., technology, design, marketing, product benefits, consumer research). These go way back to the early stages of development until the final launch of the product in the market and evaluation [1,9].

The factors influencing NPD performance are dynamic and continuously influence the project of development, so that changes in those factors must be somewhat anticipated and measured multiple times throughout a product’s life [1]. For a new product to be entirely successful, it must achieve excellence in three different areas: (a) reduced NPD cycle time, (b) high level of innovation and (c) reuse of company knowledge resources. To be successful in these three complementary areas, companies must pay attention to the factors that drive innovation: people, knowledge and systems. Product Lifecycle Management (PLM) focuses on the later (systems) and can constitute a key role for innovation and success [3,9].

## 2. Objective and Methodology

The objective of this review was to explore some aspects related to the role of consumer in the development of new foods, the factors that determine consumers’ acceptance, the innovation in traditional products, the increasing market of novel healthy foods and willingness-to-pay for innovations in food product development.

The methodology that was followed in the elaboration of this review included on a first step the selection of the topics to be addressed. For this, the specific needs of industrials and developers were taken in consideration in view of the difficulty to find information gathered about these specific issues, thus giving place to the structure of this review:

- The role of consumer;
- New foods acceptance;
- Innovation in traditional foods;
- The market of innovative functional foods;
- Willingness to pay for innovation in new product development.

Industrials and developers want to make sure that their investment in new products will pay off by revenues in sales but will depend naturally on consumer acceptance. In the second step, after establishing the studied subjects, a search was conducted on the following scientific databases: science direct, B-on, SciELO, Science Citation Index and Mendeley. For each of the topics addressed, i.e., for each of the sections in this review, appropriate keywords were used to search for relevant works. Although this was not a systematic review, some inclusion criteria were established for each of the read articles based on the relevance for the particular aspects focused in our review and the publication date as recent as possible.

## 3. The Role of Consumer

**Figure 2:**
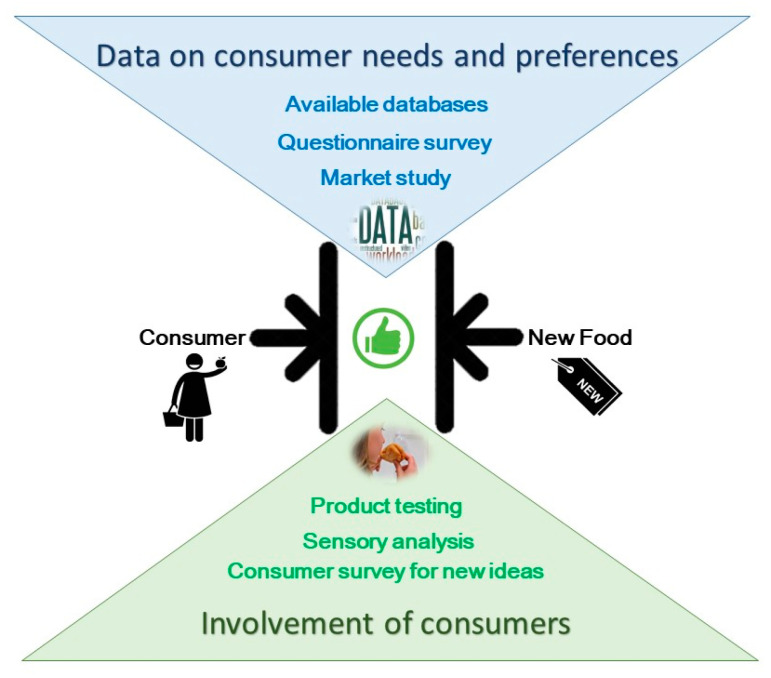
Fitting of the new food product with target consumers (author’s own work).

To assess the ideal fitting of the new product with the needs of the target consumers, there are different methods available for the food industries to rely on, such as collecting data about consumers’ needs and preferences [10,11]. A more traditional strategy includes a wide variety of tests designed to gather information about consumers’ response to new ideas and concepts of possible food products as well as concrete developed products. These allow a more directly assessment of the level of acceptance by consumers regarding those new products, so important for successful launch [11]. Other types of approach make use of indirect data, which can also be used to determine the optimal degree of fit of the new product with the expectation of consumers (Figure 2). Examples of these include data on current food trends or aggregated data on environmental factors that affect consumers’ needs and preferences, such as demographics, economical aspects, social and cultural factors or technological developments [11,12]. While the first, focusing on consumer involvement data collection and corresponding methodologies, have been more studied, the second group, namely study of consumer trends and socio-environmental factors, have been less analysed [10]. Ultimately, data obtained through direct involvement of consumers in NPD, like for example a consumer co-creation, constitute a rich source of product ideas and can have a positive impact on the successful product development and consequently improve the company’s financial performance [13,14]. Nevertheless, food firms that use food trend and socio-environmental data, which point to future changes in consumers’ needs and preferences, can more effectively develop products with longer PLC and in that way make their NPD more profitable [11,15].

Many studies have focused on consumer involvement data obtained and used only up to the launch of the new products. However, consumers’ needs and tastes change over time. Hence, the fitting of the new product with the consumer is dynamic and sometimes obliges food industries to redesign and reformulate their products, even after they had already been launched to be marketed. Even in this case, a successful redesign or reformulation must be based on knowledge regarding what consumers like, or dislike, about the existing product. Hence, it is also important to understand whether food firms obtain and employ consumer data and analyse the fitness of the new product after its launch and also during the PLC [11].

## 4. New Foods Acceptance

The study of consumer food behaviour has been based on two types of variables, i.e., some related with behavioural aspects and others linked to attitudes. While the first include measures like preference, choice, purchase or consumption, the attitudes include affective measures of the desire to select or eat foods, purchase intent or desired frequency of consumption [16,17].

Research and development (R&D) activities in the food sector should be supported by a program of research on sensory analysis and consumer acceptance of foods, and that should be well established in the company for quite some time. The NPD is supported by intrinsic as well as extrinsic factors that impact consumer acceptance, regardless of being towards conventional or novel foods. These include the role of sensorial perceptions, cognitive evaluations and situational variables [18,19].

Although the measurements of food preference and acceptance attained through attitudinal judgments can become poor predictors of consumption, owing to their degree of motivational willingness, still these types of measures continue to be used to predict consumer behaviours toward new foods, regardless of being at industrial or academic levels. This is mostly due to the easiness in assessing these measurements, in a rapid and relatively simple way, with a controlled participation of the subjects. Although with an affective nature, these evaluations in response to a tasted food have become fundamental for studying consumer behaviour towards new foods and therefore are used to orient new product development or product improvement while ensuring quality in the food industry [16].

At present, innovation practices in the food industry rely very strongly on the voice of the consumer, recognized as vital for success. Hence, strategies to develop a successful new product include an appropriate sensory evaluation allied to an understanding of the consumers’ acceptance criteria, which should be as detailed as possible [20]. When food scientists design tests intended to truthfully predict consumer behaviour at the point of purchase, they must not forget to include a proper number of variables related to marketing in their experimental design specifications, in order to guarantee that the right consumers will respond suitably to the new products [21,22]. However, for the assessment of a correct prediction of consumer behaviour, a high number of assessors is needed to evaluate food preferences regarding a specific product, which could represent a constraint.

**Figure 3:**
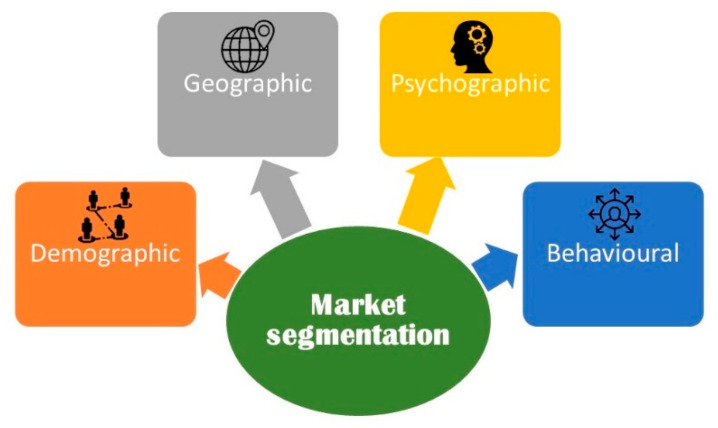
Marketing segmentation (author’s own work).

The role of the sensory analysis for success when launching a new food product is complemented with defining the target consumers. In truth, for success on the market, it is crucial to direct, eventually, the product to the right people, leading to target segmentation. Hence, food products should be market oriented according to consumers’ needs and expectations. To target a market segment, different criteria can be adopted (Figure 3): geographic variations; demographic characteristics (like sex or age); psychographic factors (including healthy or sportive lifestyles) or, lastly, behavioural criteria, like consumer’s habits and types of purchase [23].

When it comes to innovation, the food sector faces higher challenges when compared to other business areas, because people are more protective towards what they eat since, contrarily to other products, foods will enter their bodies and go all the way through the gastrointestinal tract, ending up absorbing many of their components. The concept of food neophobia, which consists in the fear of new foods, shows how this can be problematic. Although this phenomenon has been reported to have particular incidence in children, the truth is that there are people whose food neophobia persists into adulthood and truly determines their decisions when it comes to choosing between new unknown or old fully recognizable foods [17].

Consumer research and marketing dedicate attention to those segments of market interested in new products, and at the same time, the neophobic consumers shall not be neglected during the new product development process and marketing studies, because, depending on the specific product, they may represent an important share of the target market [24,25]. Some areas in which this is of particular importance include for example irradiation technology or gene mutation biotechnology, much owing to the fear of risks that these may bring for health [26,27,28,29].

The assessment of consumers’ perceptions towards foods is of vital importance in the development and marketing of new foods [30]. Therefore, understanding how consumers respond to tests helps develop effective food marketing and communication strategies. Although communication and information do not really change the characteristics of the products, they can shape the attitudes of consumers and influence their choices and behaviours [31].

## 5. Innovation in Traditional Foods

Traditional food products (TFP) have been playing an important role in European culture, heritage and identity. The growth of this segment in the European food market has been providing a higher variety of food choices for consumers [32]. Moreover, traditional food may be viewed as an opportunity to rethink rural development and sustainability patterns in many countries and to add value to the market [33,34]. There are different definitions of traditional foods in the literature that intend to capture the various dimensions of this food concept [35,36,37,38,39]. Different conceptions to define traditional food contribute to explaining consumers’ motivations to purchase traditional foods but may also cause low consumer awareness of TFPs [40]. From the general definitions, it follows that TFPs are characterized by historical, geographical and sociological dimensions. One possible definition of ‘‘traditional’’ related to foods was given by the European Commission as ‘‘traditional means proven usage in the community market for a time period showing transmission between generations; this time period should be the one generally ascribed as one human generation, at least 25 years” [41]. In 2007, the EuroFIR FP6 Network of Excellence developed an elaborative definition, which includes statements about traditional ingredients, traditional type of production and/or processing and composition [42,43]. Guerrero et al. [32] introduced in 2009 the perspective of consumers’ point of view in traditional food product definition based on a study across six European countries that analysed the data using an ordinary semantic and textual statistical on four main dimensions (habit and natural, origin and locality, processing and elaboration and sensory properties). A traditional food product, from the consumers’ perspective, was defined as ‘‘a product frequently consumed or associated with specific celebrations and/or seasons, normally transmitted from one generation to another, made accurately in a specific way according to the gastronomic heritage, with little or no processing/manipulation, distinguished and known because of its sensory properties and associated with a certain local area, region or country’’. Later, in 2010, Guerrero et al. [44] added the dimensions of health, heritage and variety to the definition of traditional foods. Furthermore, the study also notes that Central and Nordic regions tend to associate the term “traditional” primarily with practical issues such as usefulness, convenience and health whereas Southern regions tend to focus on broader concepts such as culture, heritage or history.

Although there are different definitions of TFP available in the literature, the concepts related to these food products are regulated by a European regulatory framework established in 1992 and updated in 2012 [45]. Furthermore, as part of its policy on food safety and quality and to boost competitiveness and profitability, the European Union (EU) has promoted a set of criteria for the registration and recognition of TFPs, namely, PDO (Protected Designation of Origin), PGI (Protected Geographical Indication) and TSG (Traditional Speciality Guaranteed), produced under predefined quality standards (Table 1).

Despite the controversial concept of innovation in the context of traditional foods, innovation can become an important tool to maintain and expand the market share of TFP through the improvement in convenience, safety or healthy products. Innovations in the traditional food sector have also the potential to strengthen and augment the market for traditional food products in accordance with the emerging difficulties, such as poor imitations and changing preferences and eating patterns towards more processed and convenience foods [43,47]. Other challenges such as the effective communication in the labels, legal protection of collective brands and quality assurance can contribute for the growth of traditional food market [35]. In fact, some TFPs in the EU are protected with designation quality schemes to protect producers and consumers from copycat goods. However, due to the low awareness of consumers and producers about the labels and poor understanding of the differences between them, these labels have little impact on the consumption of these traditional products. In this market, privately owned brand names are often more important quality signals to consumers than designation labels [48,49].

Innovations in traditional food are mainly introduced in the product characteristics or in packaging, which preserve the sensory quality and improve the shelf life (e.g., resealable packaging), but also in size, form and composition or in new ways of using the product but preserving the sensory quality. Given the impact of process on the authentic identity of the product, the innovation in production processes is less common and mainly refers to new technical solutions to improve quality assurance and traceability along the chain network. The organisational and market innovation can be valuable, but it is not yet recognized by all chain members of the traditional food sector and is limited to joint product development and formation of research organisations or networks [50].

For the successful introduction of innovations in traditional food products, it is also important to have a good understanding of consumers’ perceptions and attitudes towards traditional food products and of consumers’ needs and preferences when applying even small innovations to the traditional food products [51,52]. In this sense, consumers’ acceptance and improvements of traditional foods are related with product quality, innovations oriented to safer and healthier products that do not compromise their sensory properties, labels with the guarantee of origin and more product variety and convenience-oriented innovations [50]. Innovation in the traditional food sector also aims to further guarantee quality by introducing full traceability along the chain, reinforcing the message of authenticity [36]. The integration of chain partners in the innovation increases the ability to innovate while at the same time diminishes the risks involved in their implementation [53,54].

Globally, any innovation related with TFPs has to be evaluated taking into account the specifications of the product, whose market success largely depends on how consumers perceive the innovation [55].

## 6. The Market of Innovative Functional Foods

Both functional foods and nutraceuticals are food products that bear some additional health benefits beyond just nutrition. Food innovation is, among other factors, also driven by the aim to improve health or prevent disease (the scope of functional foods) or even contribute to prevent or treat certain disorders or diseases (the ambit of nutraceuticals). Although without globally accepted or legally established definitions, functional foods are recognized as providing additional benefits besides the most general functions of satisfying hunger or desire to eat and of nutrient intake [56,57].

Although there are some food categories in which more intensive development of functional foods has been made, there has been functional food development in all food categories (foods and beverages of different nature), by fortification, modification of characteristics, etc. To cite some categories in which a higher diversity and number of functional foods have been developed, one could mention the dairy sector, confectionery, soft-drinks, bakery and baby-foods [58]. Table 2 presents some relevant literature to expand the knowledge about innovations in some selected domains of functional foods.

From a product point of view, functional foods can be classified into [78,79]:

- Food fortified with additional nutrients (labelled fortified products)—example: fruit juices fortified with vitamins or dietary minerals;
- Food with additional new nutrients or components not usually present in a particular food (labelled enriched products)—example: probiotics or prebiotics;
- Food from which a harmful component has been removed, reduced or replaced by another with beneficial effects (labelled altered products)—example: use of fibres as fat releasers in high fat content foods;
- Food in which one of the components has been naturally enhanced (labelled enhanced commodities)—example: eggs with increased omega-3 content.

From the functional point of view, i.e., having in mind the objectives of the functional foods, another classification is used [80]:

- Functional foods that add benefits to life or improve children’s life—example: prebiotics and probiotics;
- Functional foods that reduce an existing health risk problem—example: foods that decrease high cholesterol or high blood pressure;
- Functional foods which make life easier—example: lactose-free or gluten-free products, for people with food allergies or intolerances.

The development of novel functional foods and nutraceuticals has been increasing largely because, on one hand, the market is demanding these products, and on the other hand, the chemistry and biochemistry of natural products as well as food technology and biotechnology have allowed important advancements. Moreover, countless studies undertaken to confirm the health claims of this type of product or their bioactive components have contributed for a great development of this market [57,81,82,83,84,85,86].

In Europe, claims of health benefits for marketing of food products are subject to Regulation (EC) No 1924/2006: Nutrition and Health Claims Made on Foods [87], which determines that any health benefits of foods announced must be scientifically proven. This regulation intends to protect consumers from deceptive or false benefits, as well as to harmonise the markets within the countries of the European Union (EU). Additionally, this regulation also aims to stimulate reliable food innovation and development. The Regulation 1924/2006 defines a health claim as any voluntary statement that refers to the relationship between food and health and establishes three classes of health claims: (1) general function claims, which can be based on generally accepted (Art. 13.1) or newly developed scientific evidence (Art. 13.5), (2) reduction of disease risk claims (Art. 14.1a) and (3) claims referring to children’s development and health (Art. 14.1b) [56,87].

Complementary health approaches include natural products and mind and body practices, as recognized by the National Centre for Complementary and Integrative Health of the USA [88]. Natural products are also considered as dietary supplements, complementary medicines, alternative medicines or traditional medicines, and are recognized by the World Health Organization as playing an important role in health promotion all over the world [89,90]. In the United States of America (USA), The Dietary Supplement Health and Education Act (DSHEA) system was introduced in 1994 to regulate supplements with health benefits [91].

In Japan, a country with a long history of utilization of foods with health benefits and the place of birth of functional foods, a functional food regulation called “foods for specified health uses” (FOSHU) was introduced in 1991. After its introduction, countless clinically proven FOSHU products with health benefits have been developed and launched in the market. Most of these products claim to be beneficial for the gastro-intestinal health, by using probiotics, prebiotics and synbiotics. Other targeted health functions with claims include lowering triglycerides level, blood pressure, LDL (low-density lipoprotein)-cholesterol and blood glucose. After 2007, in Japan, the market for FOSHU products was nearly saturated. Nevertheless, a new functional regulatory system called “Foods with Function Claims” (or New Functional Foods) was introduced in 2015, and allowed the development of many New Functional Foods due to two main reasons: higher flexibility regarding health claims as compared to FOSHU and no need for governmental approval [92,93].

Because nowadays the boundaries between the food and pharmaceutical industries are somewhat blurred, the perceptions of the consumers towards these health-related borderline products need to be investigated. The success of these products is influenced by consumers’ perception of their safety, efficacy, appearance and the placement of the product into one of those categories: food or pharmaceutical. In a survey conducted by Khedkar et al. [94] in Germany, it was found that consumers, and particularly young and highly educated women, were not convinced of the health effects of these borderline products with alleged health benefits. Although they perceived these products as GRAS (Generally Recognized as Safe), they did not consider their consumption as an easy way to stay healthy.

The attractiveness of functional foods and intention to try and eventually purchase such foods vary according to the type of product. Foods that are perceived by the consumer as healthier are judged more positively, and therefore, consumer acceptance is higher. It is important to evaluate the general principles that influence consumer responses to the health-related aspects of functional foods, including the claims, much beyond the product’s characteristics alone. One of the first aspects to consider is how believable the claims are to the consumer, since in principle, consumers frequently show some scepticism towards health or nutritional claims like those found on food labels. The construct of believability is not necessarily a strong cause for purchase intent. Still, it is supposed to interact with a number of other variables. While for some foods, the purchase intention has been influenced by low levels of scepticism towards the information provided on the product label, this is not entirely true for other foods, for which purchase intentions persist high even if the product is not fully perceived to entirely fulfil the advertised claims. Some causes that may explain this include the consumers’ familiarity with the claim and/or associated ingredients, the way in which the claim is phrased and framed, and finally the familiarity with the product itself and the extent to which the claim is consistent with the nature of the product. Another factor that influences the consumer acceptance and behaviour is the source of the claim, i.e., whether the information refers to a claim that was approved by some regulatory authority or if it is a hedonic claim made by the product manufacturer. While in many countries or regions there are strict regulations about this matter, in some others it is admissible by manufacturers to make the claims without supporting scientific evidence [95,96,97,98].

Other factors are related with the consumers’ intentions to try or purchase new foods, functional foods included, such as age, gender, education level, nutrition knowledge, dietary pattern, taste preferences or marketing and advertising. The market segmentation according to these categories, so as to consider the individual differences, has been used for long in food-related consumer research, including also the market for functional foods [98,99,100].

Lifestyle corresponds to a social concept shared by a group of people with similar attitudes towards certain variables and is strongly affected by their simultaneous needs for integration (sense of belonging) and differentiation (sense of individuality). Lifestyles of people sharing a common culture or social class and having similar professional activities are not necessarily equal. Hence, lifestyle cannot be solely attributed to demographic parameters like gender, age, education, or income but can also relate to integration or individual self-expression [98,99,100].

Consumers’ behaviours and attitudes concerning foods with health claims are also partially influenced by their own general state of health or some particular diseases, as well as the perception of how relevant the health claim might be. Additionally, the attributes that shape the perception of a food being or not helpful for a certain health/disease condition differ between groups of people [98,99,101,102].

Regarding the development of new functional foods aimed at specific markets, like older people, there are important challenges to be considered. In this ambit, care must be devoted to the sensory properties of the products, and the consumers have to be seen as particular because they show inconstant behaviours, including many of the times negative attitudes towards innovative foods or beverages [103,104]. Nevertheless, in general, it could be expected that older consumers would eventually be open to new functional foods, because these are formulated in such a way that they provide additional nutritional and health benefits, and older people tend to be more interested in maintaining health and preventing chronic diseases, when they come to a certain age where those are more probable to appear. Henceforth, to achieve success when developing new functional foods and beverages, the communication of the health benefits associated with the products must be effective to the targeted consumers [103,105,106].

Alicia et al. [107] conducted a study to understand the factors that affect consumer choice regarding foods that contain functional ingredients, by recurring to Multicriteria Decision Methods (MCDM) that are valuable to help in the decision process when designing the products in the stage of product development. The study was done with Venezuelan consumers of yoghurt, who rated with highest utility value the yogurt containing pieces of fruit, with a firm texture, which regulates intestinal function, low in fat, with sweetener (Splenda) and at an intermediate price [107].

Functional foods which increase satiety are frequently used to control appetite and help in weight loss. In order to understand the consumers’ perceptions towards this type of products, Hunter el al. [99] evaluated the influence of claims of appetite control in trustworthiness and purchasing intentions, in a sample of Australian individuals trying to lose or maintain weight. Their results showed that believability of product concept statements was highly variable, depending on the type of product. Furthermore, it was shown that consumers actively trying to lose weight demonstrated higher purchase intent as compared to consumers that were only trying to maintain their current weight, even though these two types of consumers tended to have similar levels of trust in the product concept. Variables such as age, gender or sceptical attitude towards functional foods were not found to greatly determine the purchase intent or trust regarding these functional food products [99].

## 7. Willingness to Pay for Innovation in NPD

**Figure 4:**
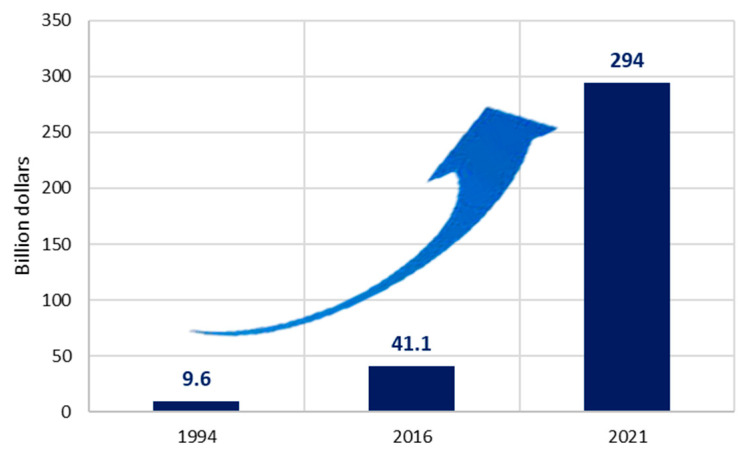
Value of the market of dietary supplements in the US [89,108].

**Figure 5:**
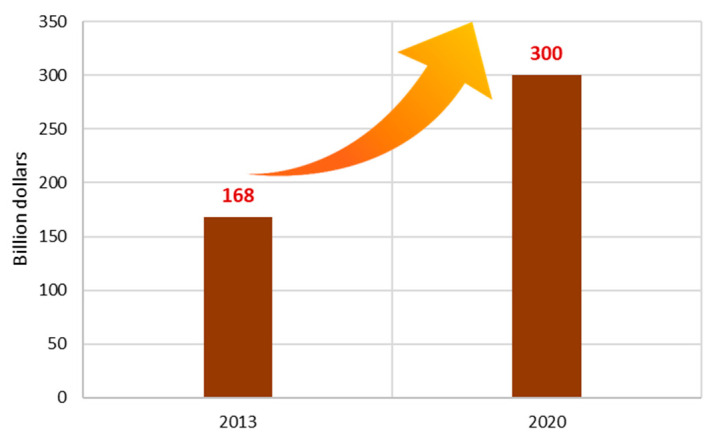
Global market value of functional foods [109,110].

It has been observed that people around the world spend a considerable amount on natural products, for example, as reported in Asia, Canada or Australia. In the USA, these natural products are bought in the form of dietary supplements, a domain in which there has been an increase in expenditure, with costs going from 9.6 billion dollars in 1994 to 41.1 billion dollars in 2016. Within the near future it is expected that over 294 billion dollars are spent on dietary supplements by 2021 [89,108] (Figure 4). The global market value for functional food products was estimated in 168 billion dollars in 2013 and can eventually nearly double in less than a decade, reaching 300 billion dollars by 2020 [109,110] (Figure 5). In japan, the total market for functional foods (FOSHU and New Functional Foods) in 2018 was 8 billion dollars [93].

The market share for functional food products is growing due to their benefits for human health and because consumers attribute increasing importance to food as part of a healthy lifestyle. Therefore, functional foods are becoming a part of consumers’ regular diets. Notwithstanding the increasing market, the rate of unsuccessful food products is high, and this trend is also observed for the market of functional food products. A high number of the newly introduced products are removed from the market soon after they are launched, and some of the possible reasons that could explain this include an overreliance on technological innovations coupled to an erroneous judgment of consumers’ needs and preferences [109,110,111,112].

According to different research findings, the willingness to purchase functional foods is influenced by factors of very diverse nature, some associated with the consumer and others with characteristics of the product, such as level of involvement of the consumer with the product, consumer lifestyle, sensory attributes and other characteristics of the product, price, brand, country of origin of the product, possible health claims announced on the packaging, the benefits of the product, among others [109].

In line with Ares et al. [113], consumers perceive functional foods as products belonging to a certain category. Hence, apart from the health benefits and the sensory characteristics, non-sensory factors like package may be determinant to shape consumers’ buying decisions. In this context, the authors studied the influence of different package attributes on consumer willingness to purchase chocolate milk desserts in two variations: the regular and an improved functional version. Their results, obtained for consumers in Uruguay, showed that consumers’ level of involvement with the product influenced their evaluation of it. Moreover, it was observed that the colour of the package and the presence of a picture on the label were the factors considered most relevant by consumers, regardless of their level of involvement with the product [113].

Romano et al. [114] investigated how Brazilian consumers perceive a non-traditional and innovative pomegranate juice by identifying the role of packaging attributes relevant to the consumer’s intention to purchase. In this study, five factors were considered: (1) the technology used in the juice production: HPP (high hydrostatic pressure) is a non-thermal technology that preserves nutritional as well as sensory properties of the products, (2) the antioxidant potential of the juice, (3) price, (4) preservatives, (5) colourings. The obtained results showed that consumers valued information about the technology used and also about the antioxidant properties, as drivers to define purchasing intentions [114].

Szakály et al. [100] evaluated to what extent lifestyle and health behaviour influenced consumption of functional foods in Hungary. The results obtained revealed that consumers make balanced decisions, looking for promotional products and bargains in order to obtain good value for their spent money. Furthermore, the authors were able to identify different segments of consumers: the rational, the uninvolved, the conservative, the careless, and the adventurous. The rational consumers, characterized by health consciousness and moderate price sensitivity, represent the most relevant target group for the functional food market. Moreover, the adventurous food consumers stand out as an equally important target group, because they are eager for novelty. The conservative consumers, characterized by positive health behaviour, also represent an interesting segment. In this way, the authors concluded that lifestyle and health behaviour are importantly linked with the preference for functional food products [100].

In a study with German consumers conducted by Goetzke and Spiller [115], it was observed that consumers who purchase functional foods and those who consume organic foods share some features regarding health and well-being. Nevertheless, they present some differentiating aspects, since the purchase of organic or functional foods is driven by different lifestyles: while purchasing of organic foods is associated to consumers with active lifestyles, the buying of functional foods is linked to consumers with a more passive lifestyle [115].

Cukelj et al. [116] studied the attitudes of Croatian consumers towards innovative flaxseed-enriched cookies, which can act as carriers for functional components like lignans and omega-3 fatty acids. The consumers revealed a high level of interest in the functional cookies, especially the elderly women with higher nutrition knowledge and consciousness [116].

Kraus [117] studied the factors influencing willingness to purchase functional foods in Poland and found that these factors were information on the health benefits and nutritional properties of the product, attributes related to taste, health and safety, practical packaging, freshness, purity and naturalness. In relation to the health benefits, the prevention of health problems and the strengthening of the body and improvement of its functions were identified as valued by consumers. With regards to the functional components, consumers showed more interest in vitamins and minerals, dietary fibre and omega-3 fatty acids. Finally, concerning the carriers, consumers preferred cereal products, dairy products, meat products and mixtures of fruits and vegetables [117].

The market of functional foods also includes products specifically addressed to children. Hence, Annunziata et al. [118] evaluated how the parents’ choices for suitable functional foods for their children are shaped, using a sample of Italian participants. The results obtained indicated that parents tend to show a strong interest in functional nutrition when choosing foods for their children, even when they are not very well familiarized with these products. Moreover, the variables that influenced the frequency of purchase were sociodemographic characteristics, parents’ nutritional knowledge, trust in those products and familiarity with them [118].

Besides the consumers’ willingness to purchase, also the willingness to pay a premium is important in new product development because it helps manufacturers to estimate the amount of profit they can expect from selling their product. This is particularly important having in mind that, nowadays, food manufacturers dedicate important budgets to R&D of new food products, as in the case of functional foods. The willingness to pay has been investigated by applying mathematical/economic models, particularly methods of experimental auction, but also contingent valuation method, choice experiment or others [109,119].

Regarding the functional foods market, it has been reported that willingness to pay can be influenced by variables such as health claims, demographic characteristics, trust in products, trust in the technologies used for their production, previous knowledge about the product or functional ingredient and degree of fitting between the carrier and the functional component [109].

Szakály et al. [120] proposed a new model for the willingness of consumers in Hungary to pay for functional foods, by modifying the Munene model. The results obtained with the modified model suggested that consumers have more positive attitudes towards functional foods and consequently are more willing to pay a premium for those products if they truly believe in their health benefits. The highest influential variables identified were the attitudes towards functional foods, followed by beliefs about the attributes of functional foods, and then by the demographic characteristics of the consumers [120].

Consumers tend to increasingly appreciate novel functional foods because they recognize their role in preventing or reducing the risks of some pathologies and particularly chronic diseases as well as improving other physiological functions, helping to achieve a better global health status. The willingness to pay for two types of functional yogurts, enriched with probiotics and with catechin, was measured by Moro et al. [119] for a sample of Italian consumers using the panel data version of a Random Parameters Logit model. The results obtained indicated that the participants were willing to pay a considerably higher premium for the catechin-enriched yogurt, almost double than that of the probiotic version. Furthermore, the results indicated that the willingness to pay for catechin enrichment was associated with grouping variables such as age, income, health status, lifestyle and education [119]. The work by Vecchio et al. [121] investigated also for Italian consumers the willingness to pay for three types of yogurt (conventional, organic and functional) considering two levels of information provided: basic (participants were presented yogurts labelled conventional, organic or functional) and advanced (participants were given additional information). The experiment was carried out using the Vickrey fifth-price sealed-bid mechanism. The findings indicated that providing additional information by, for example, a specific health claim increased consumer’s willingness to pay a premium for the functional yogurt, but a similar relation was nor observed for the organic yogurt, for which additional information on organic regulation did not add much perceived value to the extra expenditure. Moreover, it was observed that specific socio-demographic variables like gender, age, presence of children in the household and the need to follow a specific diet influence the willingness to pay for both functional and organic yogurts [121].

Romano et al. [122] used the contingent valuation method to estimate Brazilian consumer’s willingness to pay a premium for an innovative added-value pomegranate juice. The average consumer’s willingness to pay more for pomegranate juice was estimated, and the income elasticity coefficient was also calculated, so that a 10% increase in consumer income might be expected to induce a raise of about 2% in the willingness to pay the premium for the innovative pomegranate juice [122].

Roosen et al. [123] analysed how consumers trust in a new food technology, in this specific case nanotechnology. Many different concepts for trust can be found in the scientific literature, but from the economic point of view, trust can cause lower efforts towards self-protecting behaviour. Studies conducted with participants from Canada and Germany confirm that a higher level of trust in novel food products’ characteristics (orange juice) increases the willingness to pay for them, and this is also the case when new information about the technology is provided [123].

The effect of information on consumers’ preferences and willingness to pay for a functional food product (red ginseng concentrate) in Asia were investigated by Ahn et al. [124]. The results suggested that objective information can lead to discrepant changes in consumers’ valuation of different product attributes and increase willingness to pay for the functional product [124].

Pappalardo and Lusk [125] conducted a study that, based on food values and willingness to pay measures, aims to identify consumers’ subjective beliefs about functional foods. The study was performed with a sample of participants from Sicily (Italy), and the product evaluated was a new functional snack prepared with white lupine and citrus fibre. The obtained results indicated that there was a willingness to pay a premium for the product at test, and the extra value would depend both on the functional components of the product and also on other characteristics that go beyond intrinsic healthy properties. Moreover, the consumers’ willingness to pay for functional foods was clearly influenced by food values related to origin, safety, naturalness and price, among others. This means that consumers present dissimilar subjective beliefs when it comes to functional or non-functional foods [125].

Bruschi et al. [126] evaluated Russian consumers’ attitudes towards novel functional bakery products (bread and biscuits) made with purple wheat, naturally rich in anthocyanins. Because anthocyanins are a class of phenolic compounds with high antioxidant activity, they have been reported to exhibit anti-inflammatory, anti-cancer, anti-diabetic and ocular-health-enhancing properties. In this way, bakery products with high amounts of anthocyanins are considered functional foods with benefits for human health. The results obtained indicated that, despite the low level of knowledge about these bioactive compounds, when the participants were provided with information about their health-enhancing properties, most of them actually ended up valuing these products as compared with base products. Finally, the results also allowed verifying that the type of product matters (functional bread was better accepted as compared to biscuits) and the level of information provided also matters (the willingness to pay a premium was higher when information was given about the nature of the purple wheat being an old variety when compared to information about the content in anthocyanins) [126].

The ingestion of functional foods represents a somewhat inexpensive and cost-effective way to access nutritious foods that provide long-term advantages for the wellbeing of individuals and households. However, this has different impacts whether talking about urban or rural areas, in which people rely primarily, if not totally, on purchased food commodities or produced foods, respectively. The use of fortified foods or diversified/modified diets involves consuming a variety of foods that provide the diverse macro-nutrients, micro-nutrients and bioactive compounds beneficial for consumers. Nevertheless, when it comes to low income consumers, it is important to evaluate if they are willing to pay for these improved and nutritious foods, which are recommended as primary intervention to reduce nutritional deficiencies, and especially micro-nutrients, in developing countries. The work by Chege et al. [127] investigated this problematic and also how these consumers value these products, i.e., if they are accepted as the traditional basic foods or if they are regarded as luxurious new food products. For the study, they used as model a porridge flour. Their results indicated that low income consumers in Kenya and Uganda are willing to pay an extra amount for the improved biofortified porridge flour. Besides, it was observed that the willingness to pay was influenced by factors such as providing nutrition information about the product, characteristics of household, economic status of the household and presence of young children (6–59 months old) in the household [127,128].

The perception of TFPs by consumers is related to origin, locality, authenticity and gastronomic heritage of regions or countries that frequently evoke memories of childhood. Furthermore, since the TFPs are primarily appreciated by consumers for their natural nature and distinguishing sensory characteristics, the innovation of these traditional products may be accepted if it preserves the naturalness and the sensory profile of the product [39,129]. Still, other innovations that improve the healthiness and nutritional profile of TFPs are accepted by consumers, as long as they reinforce the traditional and authentic character of the product [130]. However, innovations in traditional foods seem to be more readily accepted by those who frequently consume a particular product [52]. For example, the study of Roselli et al. [55] that evaluated the willingness of consumers to accept an innovative extra virgin olive oil (EVOO) obtained by ultrasound extraction revealed that the consumers who are more keen to accept and purchase the product are those who perceived the product’s quality positively after being informed about the pivotal properties of the new product.

Pieniak et al. [131] found that while familiarity and the natural content of food is positively associated with consumption and general attitude toward traditional food, the convenience was negatively related to attitude and consumption of TFPs. However, attitude towards and consumption of TFPs was not correlated with the degree to which consumers valued sensory qualities and price sensitivity.

Molnár et al. [129] conducted a study within the European Union (EU) project TRUEFOOD (Integrated project in 6th Framework Programme; Contract no. FOOD-CT-2006-016264) to examine traditional food chain goals while also exploring the link between those and the generic consumer perceptions and choices in relation to traditional foods. In this study, the aspects identified as more relevant and important to consumers were traditionalism and quality goals.

## 8. Final Remarks

The food sector is one of the most relevant ones in the present global economy. However, companies related to food production, transformation and services still face many challenges, being one of the most pertinent the high number of unsuccessful new products. Presently, innovation practices in the food industry attribute vital importance to the voice of the consumer, recognized as essential for success. The study of consumer behaviour and attitudes towards food includes measures like preference, choice, purchase or consumption, desire to eat certain foods, buying intentions and frequency of consumption. Moreover, strategies to develop a successful new product include an appropriate sensory evaluation allied to an understanding of the consumers’ acceptance criteria. Hence, consumers can be viewed as pivotal agents to develop products with more value and able to fit market needs after launch as well as during the PLC.

Regarding the new foods’ acceptance, the incorporation of intrinsic as well as extrinsic factors on novel foods development, such as the sensorial perceptions and cognitive evaluations of consumers, are vital. Moreover, the knowledge of consumer perception helps to develop effective food marketing and communication strategies that influence their choices and behaviours.

Concerning the innovation in traditional foods, the producers still face the challenge to further improve their convenience, safety and healthiness. However, to protect the integrity of traditional products and to include the perceptions and behaviour of consumers, the innovation of TFPs should be considered in terms of specificities and with the involvement of all links in the chain of the traditional food. However, much of the development of new food products is destined to the market of functional foods and nutraceuticals, which has been increasing hugely due to higher consumer demand for these health enhancing products and because consumers are increasingly more informed. The appeal of functional foods and the intention to try and eventually purchase such foods vary according to the type of product and to how the consumer perceives their beneficial effects of their health claim.

Both the consumers’ willingness to purchase and the willingness to pay a premium are important in new product development and help food manufacturers to estimate the amount of profit they can expect by selling their products. This is so much more because nowadays food manufacturers spend important budgets on R&D of new food products, as for example in functional foods. It has been reported that the willingness to purchase functional foods is influenced by factors such as level of involvement of the consumer with the product, consumer lifestyle, sensory attributes and other characteristics of the product, price, brand, country of origin, possible health claims announced on the packaging and the benefits of the product, among others. Moreover, the willingness to pay can be influenced by variables such as health claims, demographic characteristics, trust in the products, trust in the technologies used for their production, previous knowledge about the product or functional ingredient and degree of fit between the carrier and the functional component. Concerning traditional foods, the prerequisite for consumers’ willingness to purchase and pay a premium for innovation in traditional food products is the preservation and the reinforcement of the traditional and authentic character of the products.

## Figures and Tables

**Table 1:** European Union labels for protected traditional products [46].

Protection Scheme	Symbol	Products	Specifications	Label
**Protected designation of origin (PDO)**	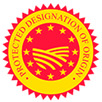	Food, agricultural products and wines.	Every part of the production, processing and preparation process must take place in the specific region. For wines, the grapes have to come exclusively from the geographical area where the wine is made.	Mandatory for food and agricultural products. Optional for wine.
**Protected geographical indication (PGI)**	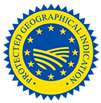	Food, agricultural products and wines.	For most products, at least one of the stages of production, processing or preparation takes place in the region. In the case of wine, this means that at least 85% of the grapes used have to come exclusively from the geographical area where the wine is actually made.	Mandatory for food and agricultural products. Optional for wine.
**Geographical indication of spirit drinks and aromatised wines (GI)**	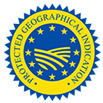	Spirit drinks and aromatised wines.	For most products, at least one of the stages of distillation or preparation takes place in the region. However, raw products do not need to come from the region.	Optional for all products.
**Traditional speciality guaranteed (TSG)**	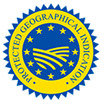	Food and agricultural products.	It highlights the traditional aspects such as the way the product is made or its composition, without being linked to a specific geographical area.	Mandatory for all products.

**Table 2:** Some relevant literature focusing on innovations in the domain of functional foods.

Category	Product	Scope	Reference
Dairy	Fermented dairy products	Scientific evidence about consumption of fermented dairy products and their health benefits	[59]
	Yogurt	Development of functional yogurts enriched with antioxidants extracted from wine	[60]
	Yogurt	Adding apple pomace as a functional ingredient to yogurt and yogurt drinks has the potential to increase the level of dietary fibre and phytochemicals, enhancing their health effects	[61]
	Dairy products	The effect of ohmic heating on probiotic metabolism and the application of this technology for the development of functional products	[62]
	Dairy products	Goat milk as a raw material for the production of functional dairy products due to the presence of functional prebiotics and probiotic bacteria	[63]
	Yogurt	Enrichment of yogurts in conjugated linolenic acid by the utilization of pomegranate and jacaranda seeds as functional components	[64]
	Yogurt	Development of yogurt formulations containing strawberries and chia seeds as health enhancing components	[65]
	Fermented dairy product	Study the feasibility for production of a functional fermented dairy-based product rich in menaquinone-7 by using iron oxide hydroxide (FeOOH) nanoparticles	[66]
Confectionary and bakery	Biscuits and breads	Use of an olive oil by-product as a functional ingredient in bakery products	[67]
	Bakery products	The use of by-products from the food industry as functional ingredients added in bakery products	[68]
	Bakery products	The use of functional ingredients in bakery products originating from marine foods	[69]
	Bakery products	The application of fibre concentrate from mango fruit as a functional ingredient with antioxidant activity in bakery products	[70]
	Biscuits and breads	Fortification of breads and biscuits with millet, oilseeds and herbs	[71]
	Bread	Fortification of bread with wheat bran protein concentrate	[72]
	Bread	Development of high-fibre wheat bread using microfluidized corn bran	[73]
Soft drinks	Plant-based dairy alternatives	Impact of the EU regulatory framework on innovation in the industry and consumers of vegetable drinks alternative to dairy products	[74]
	Fermented fruit drink	Use of coconut water and inulin as a source of soluble fibre for the development of a symbiotic fermented functional drink	[75]
	Fruit drink	The development of a functional non-alcoholic drink based on prekese fruits	[76]
	Fruit drink	Development of a functional beverage by microencapsulation of lyophilised wild pomegranate flavedo phenolics	[77]
